# Association between Thoracic Radiographic Changes and Indicators of Pulmonary Hypertension in Dogs with Heartworm Disease

**DOI:** 10.3390/ani14131900

**Published:** 2024-06-27

**Authors:** Soraya Falcón-Cordón, Yaiza Falcón-Cordón, Alicia Caro-Vadillo, Noelia Costa-Rodríguez, José Alberto Montoya-Alonso, Elena Carretón

**Affiliations:** 1 Internal Medicine, Veterinary Medicine and Therapeutic Research Group, Faculty of Veterinary Medicine, Research Institute of Biomedical and Health Sciences (IUIBS), Universidad de Las Palmas de Gran Canaria (ULPGC), 35016 Las Palmas de Gran Canaria, Spain; soraya.falcon@ulpgc.es (S.F.-C.); yaiza.falcon@ulpgc.es (Y.F.-C.); noelia.costa@ulpgc.es (N.C.-R.); elena.carreton@ulpgc.es (E.C.); 2 Department of Animal Medicine and Surgery, Faculty of Veterinary Medicine, Complutense University, 28040 Madrid, Spain; aliciac@vet.ucm.es

**Keywords:** vector-borne disease, *Dirofilaria immitis*, pulmonary hypertension, radiographic indexes, image diagnosis, echocardiography, veterinary diagnosis

## Abstract

**Simple Summary:**

Pulmonary hypertension (PH) is a high-risk condition in dogs with heartworm disease (*Dirofilaria immitis*). Echocardiography is the diagnostic technique of choice to detect PH; however, it is not accessible to all routine clinicians. Therefore, given the importance of this condition during an infection with *D. immitis*, the aim of this study was to evaluate the association of the radiological findings in dogs with heartworm disease and the presence or absence of echocardiographically characterised PH. The results obtained suggest that the evaluation of certain radiographic measures may be useful in the preliminary evaluation of the thoracic radiographs of a dog as a preliminary screening when assessing whether to perform complementary tests to evaluate the presence of PH in dogs with heartworm disease.

**Abstract:**

Pulmonary hypertension (PH) is a consequence of pulmonary endarteritis during infection with *Dirofilaria immitis* in dogs. Echocardiography is the technique of choice but is not always accessible to all clinicians. This study aimed to evaluate the association of the radiological findings in dogs with heartworm disease and the presence or absence of echocardiographically characterised PH. The study included 62 heartworm-infected dogs that underwent thoracic radiographs and echocardiography. The studied dogs showed moderate to severe PH when the Right Pulmonary Artery Distensibility (RPAD) Index was <29.5%. The RPAD Index was used for comparison with thoracic radiographs. The Vertebral Heart Size (VHS), right cranial pulmonary artery passing through the fourth rib in the laterolateral projection (CrPA/R4) ratio, and right caudal pulmonary artery to the ninth rib in the dorsoventral projection (CdPA/R9) ratio showed significant differences between dogs with/without PH (*p* < 0.001). Sensitivity (sen) and specificity (sp) cut-off values were obtained: VHS ≥ 9.53 (sen 93.75%, sp 63.33%); CrPA/R4 ≥ 1.08 (sen 87.5%, sp 70%); and CdPA/R9 ≥ 1.10 (sen 96.88%, sp 76.66%). The CrPA/R4 and CdPA/R9 ratios showed potential as a preliminary screening tool for PH in heartworm-infected dogs, suggesting that they may reliably indicate the presence of PH and guide the decision for further diagnostic testing.

## 1. Introduction

Pulmonary hypertension (PH) is one of the most common findings in dogs infected by *Dirofilaria immitis* (heartworm disease) as a consequence of the chronic development of proliferative endarteritis within the pulmonary vasculature [1]. The diagnosis of PH is mainly based on transthoracic Doppler echocardiography, which provides a non-invasive and reliable method for estimating pulmonary arterial pressure since right heart catheterisation, the gold standard for diagnosing PH, is unavailable and unacceptably invasive in compromised patients [2]. However, this method has limitations as the diagnosis is often based on indirect and subjective parameters. In addition, some of these echocardiographic measurements, such as tricuspid valve regurgitation, can be difficult to achieve. On the other hand, other estimators, such as the Right Pulmonary Artery Distensibility Index (RPAD Index), have been shown to be of great help in estimating the presence of PH [3], especially in dogs with heartworm disease [4], which can be useful in cases in which tricuspid regurgitation or pulmonary regurgitation cannot be measured.

The radiographic changes that occur in canine heartworm disease also provide important information. In infected dogs, the main findings are dilatation of the main pulmonary artery and tortuosity of the pulmonary arteries; right ventricular enlargement may also be observed in chronic infestations [5,6]. Furthermore, these signs are associated with the presence of PH in dogs with heartworm disease [7,8]. Thoracic radiography can provide supportive evidence for PH and information on concomitant or causative diseases in an individual dog [2,9], and, unlike echocardiography, this imaging technique is mostly available to the everyday clinician and does not require such specific training or dedicated equipment. Therefore, it would be interesting to perform studies aimed at evaluating its usefulness in detecting the presence of PH in this disease.

In fact, there are studies that have characterised the association of radiological and echocardiographic findings in dogs with PH [10,11], but there are not as many studies performed in dogs with heartworm disease. Given that PH is a common and serious condition in this pathology and given the unique and characteristic changes that pulmonary endarteritis produces in this pathology, this research aims to evaluate the association of the radiological findings in dogs with heartworm disease and the presence or absence of echocardiographically characterised PH.

## 2. Materials and Methods

The study included 62 dogs owned by clients who were brought to the Veterinary Service of the University of Las Palmas of Gran Canaria. The dogs lived in a hyperendemic area for *D. immitis* [12,13,14]. Inclusion in the study was based on a positive result for circulating *D. immitis* antigens (Urano test Dirofilaria^®^, Urano Vet SL, Barcelona, Spain). Dogs were also examined for the presence or absence of microfilariae using a modified Knott test. Clinical history and data including age, sex, and breed were recorded for each animal. A complete history and examination were performed on each dog to rule out the presence of other pathologies that might influence the results, and animals with concomitant diseases were excluded from the study.

On the day of diagnosis and the start of treatment (day 0), digital thoracic radiographs were taken using a radiographic unit (RX generator; model: HFQ 300 P, Bennett, NC, USA) at the time of peak inspiration without sedation. The examination protocols (kVP and mAs) were adapted specifically for each dog according to the thoracic thickness of the dog. Views were obtained in its right laterolateral and dorsoventral projections. Vertebral Heart Size (VHS) was measured according to the guidelines of Buchanan and Bücheler 1995 [15]. Although no consideration was given to excluding dogs that might have some type of alteration in the dorsal spine when interpreting VHS, none of the dogs in the present study had such alterations [16].

**Figure 1:**
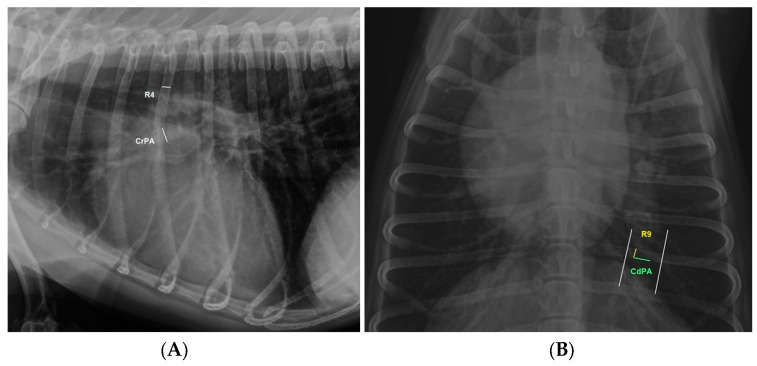
Right laterolateral (**A**) and dorsoventral (**B**) thoracic radiographs illustrating the measurement methods for this study. (**A**) The diameter of the right cranial lobar artery (CrPA) at the level of the fourth rib (R4) and the fourth rib just distal to the spine were measured, and (**B**) the diameter of the caudal lobar artery (CdPA) passing through the ninth rib (R9).

In addition, the diameter of the right cranial pulmonary artery (CrPA) passing through the fourth rib (R4) in the laterolateral projection and the diameter of R4 at a point just distal to the spine, as well as the distal and left sides of the summation shadow created by the right caudal pulmonary artery (CdPA) with the ninth rib (R9) in the dorsoventral projection, were also measured according to previous guidelines [17]. CrPA/R4 and CdPA/R9 ratios were calculated from these measurements (Figure 1). Measurements were performed using electronic callipers on a DICOM workstation (DAIPACS. 2.71 version). All measurements were performed by the same technician, blinded to the clinical status of the dogs included in this study.

Dogs underwent echocardiographic examination using an ultrasound machine with spectral and colour Doppler and multifrequency probes (5.5–10 MHz) (Logic P5, General Electric, New York, NY, USA). Dogs were placed in the right laterolateral position with the transducer in the third intercostal space. Dogs were conscious and monitored electrocardiographically throughout the study. Six continuous cardiac cycles were recorded for each measurement. All echocardiography exams were performed by the same technician. The presence or absence of PH was determined according to the American College of Veterinary Internal Medicine (ACVIM) guidelines [9]. Of all the echocardiographic indices studied, the determination of the Right Pulmonary Artery Distensibility Index (RPAD Index) was used in this study for comparison with thoracic radiographs as all dogs showed higher likelihood of moderate to severe PH when the RPAD Index was <29.5%, as previously described and validated in dogs with heartworm disease [3,4,18].

In addition, other echocardiographic findings were used to estimate worm burden [19], and a score of 1 to 4 was assigned from low to high worm burden as follows: (1) no worms visualised, (2) few worm echoes in the distal part of the right pulmonary artery, (3) worm echoes occupying the right pulmonary artery and extending to the main pulmonary artery, and (4) worm echoes occupying the entire right pulmonary artery and the main pulmonary artery to the level of the pulmonary valve. Scores of 1 and 2 corresponded to low parasite burden, and scores of 3 and 4 corresponded to high burden.

The data were analysed using the SPSS Base 29.0 software for Windows (SPSS Inc./IBM, Chicago, IL, USA). A Shapiro–Wilk test was performed to verify the normal distribution of the data. Continuous variables were expressed as median ± standard deviation. Qualitative variables were expressed as percentage. The chi-squared test or Fisher’s exact test was used to assess the association between categorical variables. In all cases, a *p* value < 0.05 was determined as significant. The results of the statistical procedures were also graphed by scatter plot. A simple linear regression was performed between the RPAD Index values and the other variables studied (VHS, CrPA/R4, and CdPA/R9 ratios) to identify the best one-variable model, and a regression analysis of all subsets was performed with a maximum improvement of R^2^ as a selection criterion. Receiver operator characteristic curve (ROC) analyses were performed to determine the optimal cut-off values for the prediction of the RPAD Index being <29.5% (moderate or severe hypertension). For all results, *p* < 0.05 was considered statistically significant. Furthermore, Cohen’s D was employed to interpret the differential magnitude between the studied statistical groups considering a statistical difference with values > 0.70 for this study.

All the owners provided their consent to participate in this study, which was carried out in accordance with the current European legislation on animal protection.

## 3. Results

Of the studied dogs, 27 were male and 35 were female, with the ages ranging from 1.5 to 12 years (mean: 5.25 years). Based on breed, 37 were mixed-breed dogs and 25 were pure-bred dogs. PH was present in 32 dogs (51.6%), with a mean RPAD Index of 29.1%. Microfilaremia was present in 40.3% of the dogs. The parasite burden was low in 87.1% of the dogs and high in 12.9% of them.

The results showed significant differences between the body weight and worm burden (*p* = 0.023), with the mean weight being 18.3 ± 10.3 kg for the dogs with a low worm burden and 11.1 ± 7.8 kg for the dogs with a high worm burden. Additionally, the dogs with PH were significantly older (*p* = 0.021).

The VHS showed a mean value of 10.1 ± 0.8 for all the studied dogs, with significant differences observed when differentiating based on the presence or absence of PH (10.41 ± 0.81 vs. 9.72 ± 0.81, respectively) (*p* < 0.001). There were no statistically significant differences in the VHS values based on the microfilaremia, parasite load, age, or sex.

The results for the CrPA/R4 and CdPA/R9 ratios were 1.21 ± 0.39 and 1.4 ± 0.5, respectively. Significant differences were observed between the groups for the CrPA/R4 ratio (1.37 ± 0.44 vs. 1.01 ± 0.23, respectively) (*p* < 0.001) and for the CdPA/R9 ratio (1.63 ± 0.56 vs. 1.16 ± 0.27, respectively) (*p* < 0.001) (Table 1). Statistically significant differences were also found for the CdPA/R9 ratio in relation to the presence or absence of microfilaremia (1.45 ± 0.35 vs. 1.37 ± 0.58, respectively) (*p* = 0.031). No significant differences were found for the rest of the studied parameters (parasite load, age, or sex).

The Pearson correlation model was used to determine whether there was a correlation between the presence of PH, based on the RPAD Index, and the studied radiographic parameters (VHS, CrPA/R4, and CdPA/R9 ratios). The correlations obtained for all three were moderately negative, indicating that, as the RPAD Index decreased, the other parameters increased. Furthermore, the results for all three correlations were statistically significant (*p* < 0.005) (Table 1).

In this study, regression analysis was performed to determine the area under the curve (AUC), coefficient of determination (R^2^), and the specificity and sensitivity of the radiographic indicators (VHS, CrPA/R4, and CdPA/R9 ratios). In addition, cut-off values were established for each of these parameters.

The CdPA/R9 ratio model proved to be the most effective in explaining the variability of the dependent variable (RPAD Index), with an R^2^ of 0.976, followed by the CrPA/R4 ratio and VHS. This indicated that CdPA/R9 had a superior ability to model the influence of the independent variables on the RPAD Index.

AUC values were calculated for each radiographic indicator and showed that all the values were above 0.5 but below 1. This suggested that the models provided a more accurate classification than would be achieved by chance, enabling the correct prediction of positive and negative cases. Specifically, the AUC for VHS was 0.78, indicating good discriminatory ability, with a 78% probability of distinguishing between the positive and negative cases. Similarly, the CrPA/R4 ratio showed an AUC of 0.77, and the CdPA/R9 ratio, the highest, reached an AUC of 0.82, highlighting its superior performance in model discrimination.

For VHS, a cut-off of 9.53 or higher resulted in a sensitivity of 93.75% and a specificity of 63.33%. This indicated a high ability to detect true positive cases, but with a moderate false positive rate. For the CrPA/R4 ratio, a cut-off of 1.08 or higher yielded a sensitivity of 87.5% and a specificity of 70%, providing a reasonable balance between detecting positive cases and minimising false positives. Finally, the cut-off for the CdPA/R9 ratio was set at 1.10 or higher, providing a sensitivity of 96.88% and a specificity of 76.66%, making it the most efficient of the three in terms of correctly identifying both positive and negative cases (Table 2).

In addition, Cohen’s D, a standardised measure of effect size, was used to interpret the magnitude of the differences between the statistical groups studied. For VHS, the calculated Cohen’s D value of 22.11 indicated a substantial difference between the dogs with the presence of pulmonary hypertension and those in absence. This extremely large effect size suggests a clinically meaningful distinction between the two groups in terms of the VHS measurements. Similarly, for the CRPA/R4 ratio, the Cohen’s D value of 4.31 indicated a significant difference between the groups. Although this is slightly smaller than the effect size for VHS, it is still a large effect size, indicating a notable difference in the CRPA/R4 ratio between the two groups. In the case of the CDPA/R9 ratio, the Cohen’s D value of 0.702, while indicating a smaller effect size compared to VHS and the CDPA/R4 ratio, still reflects a moderate difference between the dogs without PH and the group with the presence of PH. This suggests that, although the effect size was smaller, the CDPA/R9 ratio remains a relevant and potentially informative parameter to discriminate between these groups, underlining its importance in the assessment of PH (Table 3).

## 4. Discussion

Pulmonary hypertension is caused by the presence of adult *D. immitis* parasites as a result of the lesions they cause in the pulmonary arteries from the early stages of parasitism. These lesions lead to proliferative endarteritis, resulting in the enlargement, tortuosity, and loss of elasticity of the pulmonary vasculature [1]. PH is a common phenomenon in this pathology, severe and apparently irreversible in most cases [20,21], so its study and early detection should be a priority.

Echocardiography is the method of choice to detect this condition, but this technique is often inaccessible to clinical veterinarians, either due to a lack of knowledge or equipment, or to animal owners due to financial constraints. However, radiology is a diagnostic imaging technique widely used in all veterinary clinics that is less technically demanding and more affordable [22]. Therefore, in this study, the authors aimed to evaluate its usefulness as a first approximation imaging tool to determine the presence or absence of PH in dogs with heartworm disease.

The results showed significant differences between the VHS indices and the CrPA/R4 and CdPA/R9 ratios in animals with PH compared with those without PH in dogs with heartworm disease. Previous studies have demonstrated radiological changes in dogs with PH caused by several pathologies, mainly cardiomegaly, with other findings described as infiltration of the lung parenchyma and enlargement of the pulmonary arteries [10,23]. In addition, the ECVIM guidelines recommend that echocardiography be performed when a dog has radiological changes consistent with PH [9]. Indeed, thoracic radiography is a useful technique for detecting cardiopulmonary abnormalities in dogs with *D. immitis*, with the enlargement of the right ventricle, the main pulmonary artery, and the right lobar pulmonary artery being the most commonly reported abnormalities [24,25,26]. However, no studies were found on the use of radiological changes objectively as an estimator of PH, so the results of this study validate the clinical usefulness of radiographs in dogs with heartworms to estimate the presence of PH.

The results obtained showed that the VHS was increased in the group with PH compared to the group without PH. Other authors have reported increased cardiac silhouettes on thoracic radiographs in dogs with PH, especially when symptomatic [25,27]. The cut-off value of 9.53 provides high sensitivity but low specificity for the detection of PH, which is consistent as the value of 9.53 is within the normal range for healthy dogs [15]. Therefore, although statistically there are differences between VHS as a function of the presence or absence of PH, and there is a significant correlation between the RPAD Index and VHS, it could not be considered an adequate value to determine the presence of this condition with the results of this study. In dogs with heartworm disease, cardiomegaly occurs only in the final stages of the disease and in dogs with severe PH, whereas this study included dogs with PH considered to be moderate to severe.

The comparison between the pulmonary arteries and the rib is a useful and common criterion to assess the pulmonary vasculature in dogs, as pointed out by previous authors [17,28], also in canine heartworm disease [24,25]. In *D. immitis* infection, there is enlargement, dilation, and increased tortuosity of the pulmonary arteries as a result of endarteritis, leading to PH. However, whether these vascular changes can act as predictors of PH in infected dogs has never been investigated. The results showed that both ratios showed significant differences between the presence/absence of PH. Furthermore, there was a correlation between both ratios and the RPAD Index, especially for the CdPA/R9 ratio. The cut-off values for the CrPA/R4 (≥1.08) and CdPA/R9 (≥1.10) ratios showed good sensitivity, excellent in the case of the CdPA/R9 ratio, with good specificity, indicating that these parameters could be used in the radiological evaluation of dogs with heartworm disease as a preliminary screening to determine the need for further testing for the presence of PH. These cut-off values are different from the reference values established by previous authors for the CrPA/R4 ratio < 1.2 [28] and CdPA/R9 of 1 for healthy dogs [29,30], or those described to differentiate dogs with mitral regurgitation from healthy dogs (CrPA/R4 (≥0.95) and CdPA/R9 ≥ 1.32) [17].

Other authors have already demonstrated the usefulness of these radiological indices to detect PH caused by other thoracic pathologies, such as Oui et al. (2015) [17], who used these indices, among others, to differentiate between dogs with mitral regurgitation and healthy dogs. In addition, other authors have demonstrated the usefulness of other similar radiological indicators to predict the presence of PH caused by different pathologies, such as the ratio of the area of the pulmonary artery crossing the ninth rib to the area of the ninth thoracic vertebra (areaPA/areaT9), the ratio of the width of the pulmonary artery crossing the ninth rib to the width of the ninth thoracic vertebra (widthPA/widthT9), or the caudal pulmonary artery to vein ratio [11,31].

As a limitation of the study, it must be remembered that, in veterinary medicine, the measurement of PH is based on indirect echocardiographic measurements and that they have not been confirmed with direct measurements through right heart catheterisation.

## 5. Conclusions

In conclusion, an increase in the cardiac silhouette does not appear to be useful in assessing the presence or absence of PH in dogs with heartworm disease. However, the results obtained for the CrPA/R4 and CdPA/R9 ratios seem to show cut-off values with quite acceptable sensitivity and specificity, which could suggest the evaluation of these ratios when carrying out a preliminary evaluation of the thoracic radiographs of a dog as a preliminary screening when assessing whether to perform complementary tests to evaluate the presence of PH. Moreover, additional studies with a larger number of animals, to enable a more robust statistical analysis, are necessary to further evaluate these radiological indicators.

## Figures and Tables

**Table 1:** Correlation coefficients for all studied radiographic parameters.

Correlation	Coefficient	Interpretation
IDAPD-VHS	−0.4776417 ***	Moderate negative correlation
IDAPD-CRPA/R4	−0.4344274 ***	Moderate negative correlation
IDAPD-CDPA/R9	−0.53781329 ***	Moderate negative correlation

*** Correlation is significant at 0.5% (*p* < 0.005).

**Table 2:** Results of simple regression analyses for the prediction of PH using the Right Pulmonary Artery Distensibility Index (RPADI < 29.5%). R^2^ (coefficient of determination); AUC (area under receiver operating characteristic curve); VHS (Vertebral Heart Size); CrPA/R4 (right cranial pulmonary artery passing through the fourth rib in the laterolateral projection ratio); CdPA/R9 (right caudal pulmonary artery to the ninth rib in the dorsoventral projection ratio).

Parameter	R^2^	AUC	Cut-Off Value	Sensitivity	Specificity
VHS	0.853	0.78	≥9.53	93.75%	63.33%
CRPA/R4	0.959	0.77	≥1.08	87.5%	70%
CDPA/R9	0.976	0.82	≥1.10	96.88%	76.66%

**Table 3:** Cohen’s D for the three studied parameters. VHS (Vertebral Heart Size); CrPA/R4 (right cranial pulmonary artery passing through the fourth rib in the laterolateral projection ratio); CdPA/R9 (right caudal pulmonary artery to the ninth rib in the dorsoventral projection ratio).

Measure	Cohen’s D	Interpretation
VHS	22.1	Large effect
CrPA/R4	4.31	Large effect
CdPA/R9	0.702	Moderate effect

Cohen’s D: d = 0.2 small effect; d = 0.5 moderate effect; d = 0.8 large effect.

## Data Availability

All data generated or analysed during this study are included in this article. The datasets used and/or analysed during the present study are available from the corresponding author upon reasonable request.
